# Strain and Sex Variability in Liver, Kidney and Lung Levels of DNA Adducts EB-GII and *bis*-N7G-BD Following Inhalation Exposure to 1,3-Butadiene in Collaborative Cross Mice

**DOI:** 10.3390/toxics13100844

**Published:** 2025-10-03

**Authors:** Erik Moran, Samantha Goodman, Fred A. Wright, Richard Evans, Natalia Y. Tretyakova, Ivan Rusyn

**Affiliations:** 1 Department of Medicinal Chemistry and Masonic Cancer Center, University of Minnesota, Minneapolis, MN 55455, USA; moran598@umn.edu (E.M.); evan0770@umn.edu (R.E.); 2 Department of Veterinary Physiology and Pharmacology, College of Veterinary Medicine and Biomedical Sciences, Texas A&M University, College Station, TX 77843, USA; goodman.samantha.j@gmail.com; 3 Bioinformatics Research Center and Departments of Biological Sciences and Statistics, North Carolina State University, Raleigh, NC 27695, USA; fred_wright@ncsu.edu

**Keywords:** 1,3-butadiene, DNA adducts, mouse model, intra-species variability

## Abstract

1,3-butadiene (BD) is a volatile organic pollutant. Upon inhalation, it is metabolically activated to reactive epoxides which alkylate genomic DNA and form potentially mutagenic monoadducts and DNA–DNA crosslinks including N7-(1-hydroxyl-3-buten-1-yl)guanine (EB-GII) and 1,4-*bis*-(guan-7-yl)-2,3-butanediol (*bis*-N7G-BD). While metabolic activation resulting in mutagenicity is a well-established mode of action for 1,3-butadiene, characterization of the extent of inter-individual variability in response to BD exposure is a gap in our knowledge. Previous studies showed that population-wide mouse models can be used to evaluate variability in 1,3-butadiene DNA adducts; therefore, we hypothesized that this approach can be used to also study variability in the formation and loss of BD DNA adducts across tissues and between sexes. To test this hypothesis, female and male mice from five genetically diverse Collaborative Cross (CC) strains were exposed to filtered air or 1,3-butadiene (600 ppm, 6 h/day, 5 days/week for 2 weeks) by inhalation. Some animals were kept for two additional weeks after exposure to study DNA adduct persistence. EB-GII and *bis*-N7G-BD adducts were quantified in liver, lungs and kidney using established isotope dilution ESI-MS/MS methods. We observed strain- and sex-specific effects on both the accumulation and loss of both DNA adducts, indicating that both factors play important roles in the mutagenicity of 1,3-butadiene. In addition, we quantified the intra-species variability for each adduct and found that for most tissues/adducts, variability values across strains were modest compared to default uncertainty factors.

## 1. Introduction

1,3-butadiene (BD) is an important industrial and environmental chemical that has been implicated in several diseases including cancer in humans and animals [1,2]. BD is present in tobacco smoke [3], wood smoke [4], cooking vapors [5,6], and tailpipe emissions [7], and is among the most hazardous chemicals an average person is routinely exposed to in an urban environment [8]. Animal studies have confirmed that BD represents a potent health risk—it has been shown to cause mutations and tumors in laboratory animals [9,10] and is classified as a known human carcinogen [2]. In 2024, the United States Environmental Protection Agency concluded that BD “*presents an unreasonable risk of injury to human health for workers and the general population (including fence line communities) from inhalation exposure [and that BD] is associated with health effects such as reduced birthweight pregnancies, blood and immune system disease, and leukemia*” [11]. Because of its potent ability to induce tumors in laboratory animals and the widespread human exposure to BD, it is critical to characterize the extent of inter-individual variability in response to BD.

**Figure 1:**
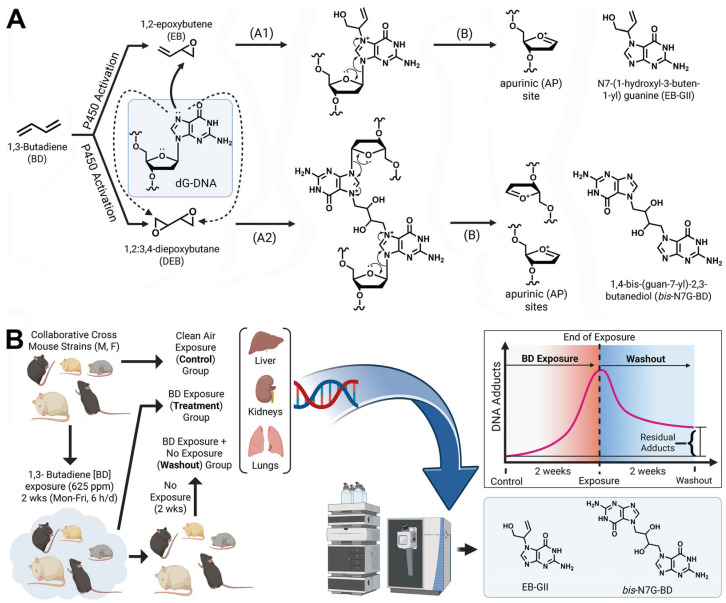
(**A**) 1,3-Butadiene (BD)-DNA adduct formation via metabolic activation to DNA-reactive epoxides produces EBGII (A1) and *bis*-N7G-BD (A2). N-7-guanine adducts are released from DNA via neutral thermal hydrolysis generating AP sites (B). (**B**) Overall study design to quantify BD-induced DNA adduct formation and loss in five genetically diverse Collaborative Cross mouse strains.

Following inhalation exposure, BD is metabolized to 3,4-epoxy-1-butene (EB) and 1,2,3,4-diepoxybutane (DEB) via metabolic oxidation catalyzed by cytochrome P450 monooxygenase 2E1 (Figure 1A) [12,13]. If not detoxified via hydrolysis or conjugation with glutathione, these reactive epoxides can alkylate cellular DNA and proteins to form covalent adducts. If formed in genomic DNA, nucleobase BD adducts can destabilize DNA structure, induce strand breaks, and cause mutations by compromising the fidelity of DNA polymerases [14,15]. Therefore, BD DNA adducts are useful mechanism-based biomarkers of butadiene exposure and potential cancer risk [13]. BD DNA adducts formed at the N7 position of guanine are by far the most abundant and can be removed via spontaneous depurination and apurinic site repair (Figure 1A). BD-modified DNA bases such as EB-GII and *bis*-N7G-BD have been quantified in tissues and urine of exposed laboratory animals [16].

The biological response to BD and the ultimate impact of BD exposures can be highly variable amongst genetically diverse human populations where bioactivation, repair, and detoxification differ due to genetic variation [17]. For example, cancer risk in smokers varies by ethnicity, with African Americans and Native Hawaiians being at a higher risk for lung cancer development, which could be explained by inter-individual differences in response to BD and other tobacco carcinogens [18]. It is important to understand both the mechanisms and extent of inter-individual variability in response to environmental and lifestyle hazardous chemicals. To demonstrate genetic variability in animals, panels of recombinant inbred strains have been used to evaluate intra-species genetic variation in controlled exposure studies such as the mouse diversity panel [19], BXD [20], LXS [21], and Collaborative Cross (CC) [22]. Several of these population-wide models have been previously employed to study genotoxic and epigenetic effects of BD [16].

The present study utilized a genetically diverse CC mouse population [22] to investigate BD DNA adduct formation and persistence across different strains, genders, and tissues. A highly sensitive and specific isotope dilution HPLC-ESI-MS/MS methodology [16] was employed to measure the formation of N7-(1-hydroxyl-3-buten-1-yl)guanine (EB-GII) and 1,4-bis-(guan-7-yl)-2,3-butanediol (*bis*-N7G-BD) in five CC strains of mice that were selected from a larger panel to represent the spectrum of response to BD in genetically diverse populatione [23]. Mice were exposed to 600 ppm of BD or filtered air by inhalation (Figure 1B). To study the persistence of DNA adducts, additional mice from each exposed group were kept in BD-free conditions for 2 weeks post-exposure, and residual levels of EB-GII and *bis*-N7G-BD in tissues were measured. Variability in DNA formation and loss was quantified to inform intra-species variability assumptions for BD risk assessment [11].

## 2. Materials and Methods

### 2.1. Chemicals

Chemicals and solvents including LC–MS-grade acetic acid, water, methanol, and acetonitrile were acquired from Fisher Scientific (Pittsburgh, PA, USA). DNA isolation materials were acquired from Qiagen (Hilden, Germany). Glass vials (300 μL) were acquired from ChromTech (Apple Valley, MN, USA). EB-GII and ^15^N_5_-EB-GII, *bis*-N7G-BD, and ^15^N_6_-*bis*-N7G-BD standards were synthesized in our laboratory as reported previously [24]. *bis*-N7G-BD and ^15^N_6_-*bis*-N7G-BD standards were synthesized as previously described. Concentration and purity of *bis*-N7G-BD standards were previously established [25]. DNA adduct standard stock solutions were prepared in sterile water and stored at −20 °C.

### 2.2. Animal Study and Tissues

The in-life portion of the study was previously described [16]. Briefly, male and female mice (aged 10–15 weeks before the start of exposures) from five CC strains (CC026, CC027, CC043, CC049, CC079) were obtained from the UNC Systems Genetics Core (Chapel Hill, NC, USA). Strains for this study were selected based on the availability of sufficient numbers of same-age males and females and represented a range of genotoxic effects of BD (e.g., the formation of various BD adducts) as reported in a previous study of 20 CC strains [23]. Female mice were group-housed by strain, while males were housed individually, all in temperature-controlled, pathogen-free conditions with a 12 h light/dark cycle. Mice were given standard chow and purified water ad libitum and acclimated for 30–45 days before the study. Mice were randomized by weight and sex into three groups (filtered air and two BD exposure groups, five mice per group). Whole-body exposures occurred for 6 h/day, 5 days/week, over two weeks at a concentration of 590 ± 150 ppm, a level that is relevant to prior mouse studies of BD-induced toxicity and tumorigenesis.

During exposures, mice were placed in mesh holders within sealed chambers with monitored airflow. Two chambers were injected with BD and the third one was flushed with filtered air. Air samples were analyzed every 10–15 min via gas chromatography. After each 6 h exposure period, chambers were flushed with clean air, and mice were returned to individual cages overnight. Cotton pads and the mesh holders were added to the cages to reduce stress. Exposures for males and females were run in separate two-week periods, alternating groups daily between exposure chambers to equalize conditions. Animal health was monitored multiple times per day before and during exposure. Moribund animals were sacrificed with Euthasol upon consultation with the staff veterinarian in the animal facility. Mice were weighed twice weekly. At the end of the 2 week exposure period, necropsies and tissue collections were performed within 2 h of the cessation of exposure. Specifically, mice from the filtered air (Control) and BD-exposed groups (Treatment) were euthanized by exsanguination following deep isoflurane anesthesia. Tissues and blood were removed, snap-frozen in liquid nitrogen, and stored at −80 °C. One of the BD-exposed cohorts (Washout) was kept for 2 additional weeks without exposure in a manner similar to the pre-exposure housing detailed above. These animals were euthanized and organs were removed as detailed above. All experimental procedures involving animals and their husbandry were approved by the Institutional Animal Care and Use Committee of Texas A&M University (protocol IACUC 2020-0271).

### 2.3. Genomic DNA Extraction and Quantitation

DNA was isolated from frozen tissues using a previously described method with the following modifications to enhance purity. Initially, 50–100 mg of tissue (liver, kidney or lung) was homogenized into 6 mL of cell lysis solution (Qiagen) using a TissueRuptor II (Qiagen), followed by the addition of 30 μL of Puregene Proteinase K solution (Qiagen). Samples were incubated overnight to achieve cell lysis, followed by incubation with 30 μL of Puregene RNase A solution (Qiagen) for 2 h for RNA digestion. Proteins were precipitated with 2 mL of protein precipitation solution (Qiagen), followed by centrifugation at 2000× *g* for 15 min. DNA was precipitated with 10 mL of ice-cold isopropyl alcohol and stored at −20 °C overnight, followed by centrifugation at 2000× *g* to pellet DNA. Pelleted DNA was reconstituted in 1 mL of 10 mM of Tris-HCl (pH 7.5) and subjected to a second incubation with 30 μL of RNase A solution (Qiagen) for 2 h. RNase A was precipitated via the addition of 750 μL of Protein Precipitation Solution (Qiagen), followed by centrifugation at 2000× *g* for 15 min. A 24:1 mixture (1.75 mL) of chloroform and isoamyl alcohol (Sigma-Aldrich, St. Louis, MO, USA) was added to each sample, vortexed for 20 s, and centrifuged at 3100× *g* for 15 min. The upper layer containing DNA was removed, and DNA was precipitated with the addition of ice-cold isopropyl alcohol (4 mL). DNA was washed with 70% ethanol in water (1 mL) and 100% ethanol (1 mL).

DNA was quantified utilizing enzymatic digestion and HPLC-UV analysis of dG as previously described with the following modifications to improve method throughput. DNA digests were performed in 2 mL 96 deep-well plates combining DNA, phosphodiesterase I and II (Worthington Biochemical, Lakewood, NJ, USA), DNaseI (Worthington Biochemical), and alkaline phosphatase (Sigma-Aldrich). Overnight incubation was performed in a 37 °C water bath. Enzymes were removed by vacuum filtration via a 96-well 10K nanosep filter plate (Pall, Port Washington, NY, USA). The resulting filtrate was analyzed directly by injection to HPLC-UV system (Agilent, Santa Clara, CA, USA) with a gradient of 5 mM of NH_4_HCO_2_ (pH 4) (A) and methanol (B) adjusted linearly from 3% B to 30% B in 15 min flowing at 0.9 mL/min using an Atlantis T3 column (4.6 × 150 mm, 3 µm) (Waters, Milford, MA, USA) with dG eluting at ~10 min. dG concentrations were calculated against a standard curve of dG prepared from 17 to 177 µM analyzed by the same HPLC-UV method.

### 2.4. Neutral Thermal Hydrolysis and HPLC Offline Enrichment and Nano-LC-HRMS Analysis of EB-GII and bis-N7G-BD DNA Adducts

**Figure 2:**
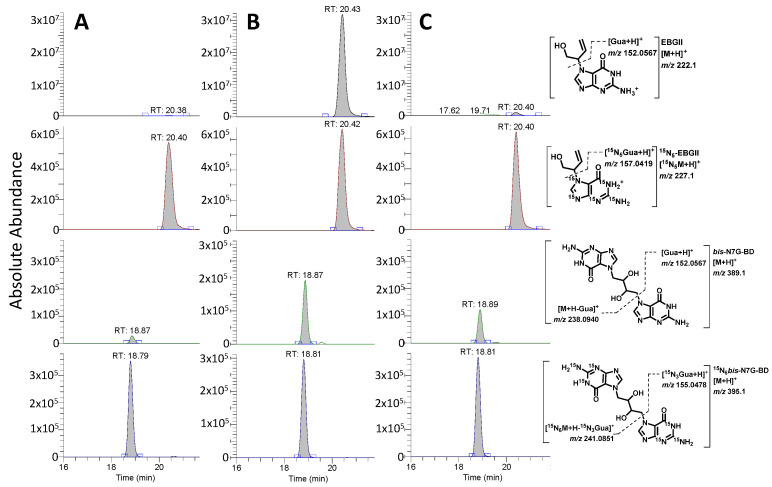
Representative nano-LC-ESI-MS/MS traces for quantification of EB-GII (top two traces) and *bis*-N7G-BD (bottom two traces) in liver samples of male CC049 mice exposed to clean air (**A**), 600 ppm BD (**B**), or allowed to recover for 2 weeks following exposure to BD (**C**).

DNA isolated from tissues was concentrated under a vacuum and spiked with ^15^N_5_-EB-GII and ^15^N_6_-*bis*-N7G-BD internal standards (Figure 2A). Spiked DNA was subjected to neutral thermal hydrolysis and filtration to remove DNA backbone and unadducted DNA as previously described. Filtrates were enriched for EB-GII and *bis*-N7G-BD using a HPLC-UV system (Agilent) equipped with an automated fraction collector. A gradient of 0.4% (*v*/*v*) formic acid in water (A) and acetonitrile (B) at 1 mL/min was applied to samples injected on a Zorbax XDB-C18 column (4.6 × 150 mm, 5 µm) (Agilent) with a combined fraction for EB-GII and *bis*-N7G-BD eluting at ~17 min. HPLC fractions were dried completely under reduced pressure or under a gentle nitrogen stream and reconstituted by sonication in LC-MS-grade water for nano-LC/HRMS analysis on a QExactive Orbitrap mass spectrometer coupled to a Dionex UPLC system as previously described [25] with additional parallel reaction monitoring transitions for EB-GII (222.1 → 152.0567) and ^15^N_5_-EB-GII (227.1 → 157.0419) (Figure 2A). Concentrations of EB-GII and *bis*-N7G-BD were calculated by the comparison of area ratios of peaks corresponding to the analytes and known amounts of internal standards, which were added prior to neutral thermal hydrolysis. Sample concentrations of EB-GII and *bis*-N7G-BD were converted to adducts per 10^6^ (for EB-GII) or 10^7^ (for *bis*-N7G-BD) nucleotides using DNA amounts obtained from dG analysis. To validate the sensitivity of the method, the limit-of-detection (LOD) and limit-of-quantitation (LOQ) were evaluated by spiking a non-contributing sample matrix (salmon sperm DNA, Sigma-Aldrich) with equal levels of internal standard (2 fmol) and 0–40 fmol of unlabeled standard. Using 5 replicates for each validation concentration, these samples were subjected to the described standard sample preparation and analysis. LOD/LOQ values for EB-GII and *bis*-N7G-BD (1.03/3.10 and 1.62/4.85 adducts/10^9^ nucleotides, respectively) were determined by the formulae 3.3σ/*S* and 10σ/*S*, where σ is the standard error of the slope and *S* is the slope of the validation curve for each analyte (see Supplementary Figure S1). To account for measurements <LOD and <LOQ, respectively, values of LOD/2 and LOQ/2 were substituted to facilitate for statistical comparison of treated animals to controls.

### 2.5. Statistical Analyses

**Figure 3:**
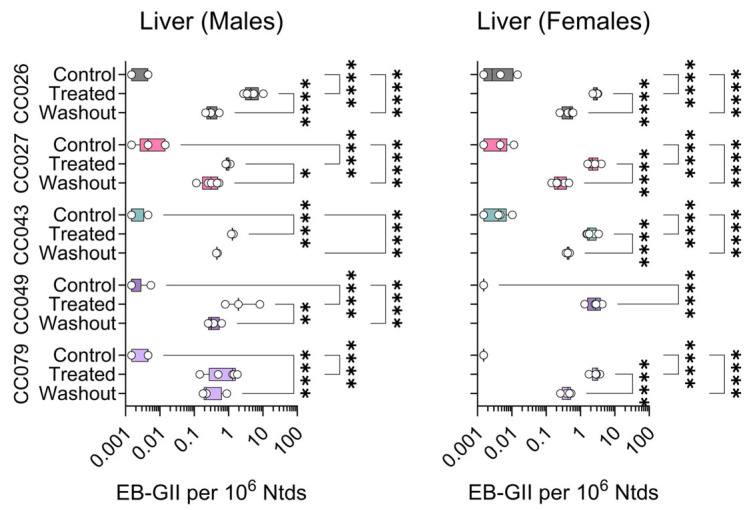
Strain- and sex-specific levels of EB-GII adducts in the livers of Collaborative Cross mice treated with clean air (Control), 1,3-butadiene (600 ppm for 2 weeks, Treated), or left unexposed for 2 weeks after 1,3-butadiene treatment (Washout). Shown are box (inter-quartile range)-and-whisker (min to max values) plots that also include all individual data points. See the Methods Section for details on statistical comparisons. No washout samples were available for analysis from CC049 female mice.

**Figure 4:**
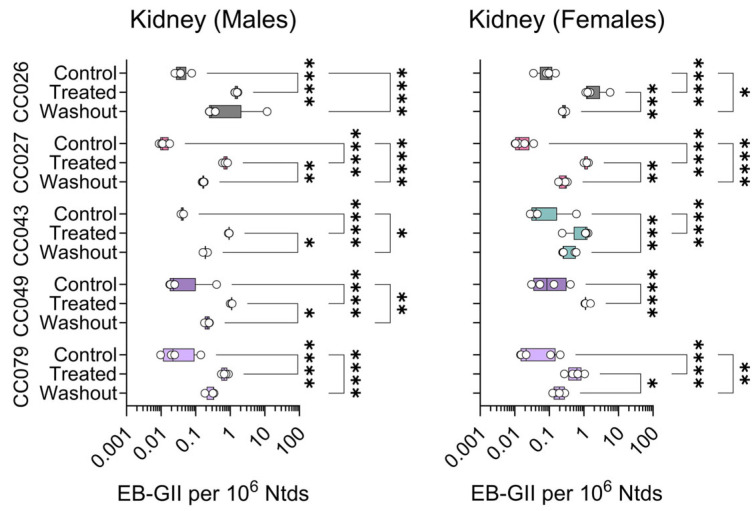
Strain- and sex-specific levels of EB-GII adducts in the kidneys of Collaborative Cross mice treated with clean air (Control), 1,3-butadiene (600 ppm for 2 weeks, Treated), or left unexposed for 2 weeks following 1,3-butadiene treatment (Washout). Shown are box (inter-quartile range)-and-whisker (min to max values) plots that also include all individual data points. See the Methods Section for details on statistical comparisons. No data is shown for CC049 washout female mice because samples were unavailable.

**Figure 5:**
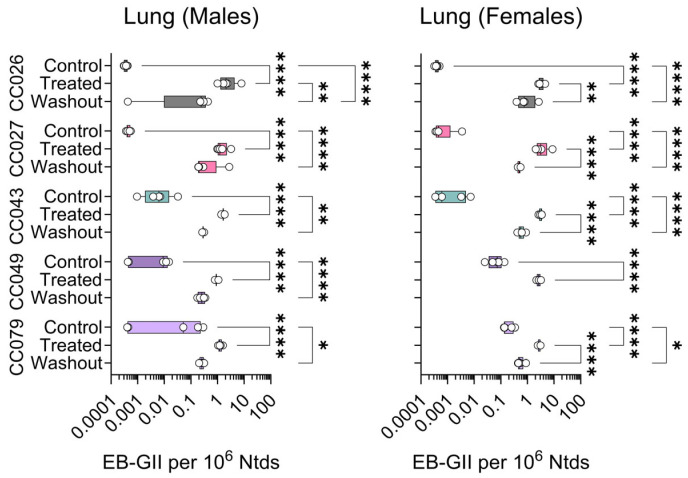
Strain- and sex-specific levels of EB-GII adducts in the lungs of Collaborative Cross mice treated with clean air (Control), 1,3-butadiene (600 ppm for 2 weeks, Treated), or left unexposed for 2 weeks after 1,3-butadiene treatment (Washout). Shown are box (inter-quartile range)-and-whisker (min to max values) plots that also include all individual data points. See the Methods Section for details on statistical comparisons. No data is shown for CC049 female mice because samples were unavailable.

**Figure 6:**
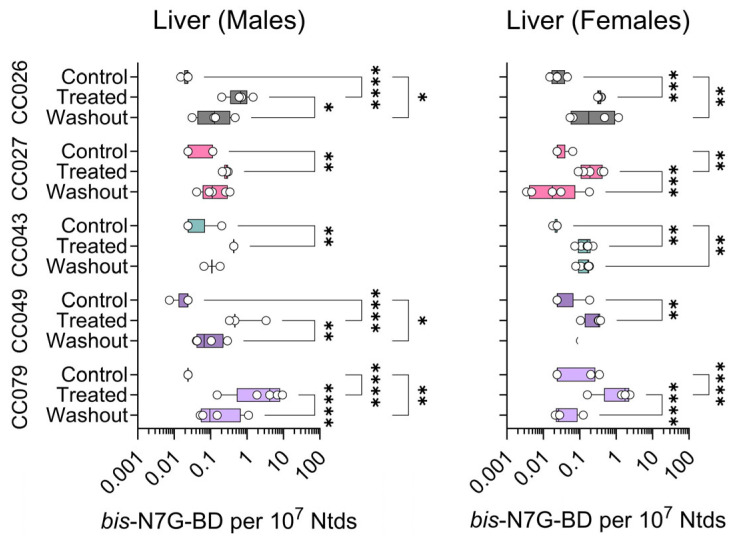
Strain- and sex-specific levels of *bis*-N7G-BD adducts in livers of Collaborative Cross mice treated with clean air (Control), 1,3-butadiene (600 ppm for 2 weeks, Treated), or left unexposed for 2 weeks after 1,3-butadiene treatment (Washout). Shown are box (inter-quartile range)-and-whisker (min to max values) plots that also include all individual data points. See the Methods Section for details on statistical comparisons. No data are shown for CC049 female mice.

Adduct values were primarily analyzed after logarithmic transformation, except for geometric means and standard deviations reported in Table 1. Analyses were performed in GraphPad Prism (version 10.3.1, GraphPad Software, San Diego, CA, USA) and *R* version 4.4. For the data shown in Figure 3, Figure 4, Figure 5 and Figure 6, differences between groups were evaluated using 2-way analysis of variance (ANOVA) on Log_10_-converted data with Tukey’s correction for pairwise comparisons. Significant differences are indicated by asterisks (*p*-values were multiplicity-adjusted to account for multiple comparisons) as follows: *, *p* < 0.0332; **, *p* < 0.0021; ***, *p* < 0.0002; ****, *p* < 0.0001. Additional ANOVA/regression analyses were performed for the BD exposure condition and the combined exposure/washout conditions to evaluate sex/strain effects, and the degree of recovery following exposure. These analyses included two-way interactions, and Bonferroni multiple-comparison *p*-value correction for each analysis, with a significance level of 0.05.

Estimates of variability across/within strains and uncertainty factors were conducted for exposure and washout conditions separately, and closely followed procedures detailed elsewhere [25]. Adducts were analyzed on the natural log scale, and after exponentiation are presented as geometric means and standard deviations. Mixed linear models, with sex as a fixed effect and strain as a random effect, provide directly interpretable population variability estimates. The R package lme4 (version 1.1-37) was used to provide restricted maximum likelihood estimates of the strain variability component, with profile likelihood used to provide confidence intervals for variability. We computed σ as the standard deviation of mean log adduct values across strains, and uncertainty factor high (UF_H_) values, which are protective upper limits, as exp(z_0.95_ σ) and exp(z_0.99_ σ) for the 95th and 99th percentile values.

## 3. Results

Our approach for accurate quantification of BD DNA adducts in tissues of animals exposed to BD employed neutral thermal hydrolysis to selectively release EB-GII and *bis*-N7G-BD adducts as free bases from the DNA backbone (see Figure 1A). This method is analogous to the previously reported methodology for genomic EB-GII and urinary *bis*-N7G-BD adducts [23] but streamlined sample processing for higher throughput by simultaneous enrichment and analysis of EB-GII and *bis*-N7G-BD. Following the addition of isotopically labeled internal standards, DNA samples were heated to release EB-GII and *bis*-N7G-BD as free bases, and the remaining high-molecular-weight DNA was removed by ultrafiltration. Next, EB-GII and *bis*-N7G-BD were enriched by offline HPLC and analyzed using a high-resolution Orbitrap Q Exactive mass spectrometer. Sample processing employed here allowed for matrix simplification and ultimately increased sensitivity for the LC-MS analysis of EB-GII and *bis*-N7G-BD. Representative nano-LC-ESI^+^ HRMS/MS traces for control, BD-exposed, and mice allowed to recover for 2 weeks post-exposure (Washout) samples are shown in Figure 2. As seen in the extracted ion chromatograms for each sample, all analytes were monitored for loss of the guanine substituent from protonated molecules of the analyte at *m*/*z* 222.1 (EB-GII), 227.1 (^15^N_5_-EB-GII internal standard), 389.1 (*bis*-N7G-BD), and 395.1 (^15^N_6_-*bis*-N7G-BD internal standard), resulting in product ions corresponding to protonated guanine (*m*/*z* 152.0567 for EB-GII and 157.0419 for ^15^N_5_-EB-GII ) or monoalkylated-guanine at *m*/*z* 238.0940 for *bis*-N7G-BD and 241.05851 for the ^15^N_6_-*bis*-N7G-BD internal standard (Figure 2).

Employing this sensitive analytical method, DNA isolated from liver, kidney and lung samples from Collaborative Cross mice exposed to BD or clean air were analyzed to quantify genomic DNA adducts EB-GII and *bis*-N7G-BD. Figure 3 shows the data for EB-GII in the liver samples. In male mice, BD adduct levels were significantly higher in exposed animals compared to controls, and some variability was observed within each strain. In the washout group, levels of this adduct significantly declined from levels in animals exposed to BD for 2 weeks in CC026, CC027, and CC049 strains. In females, EB-GII levels significantly increased upon exposure for all strains; similarly, in all strains, adduct levels in the washout samples were significantly lower than those detected immediately after 2 weeks of exposure (Figure 3). No washout samples were available for analysis from CC049 female mice.

For both kidney (Figure 4) and lung (Figure 5) DNA, EB-GII adduct levels were slightly lower than those in the liver (Figure 3). In the kidney, adduct levels were significantly increased with treatment in both sexes and all strains. Treatment effects were significant only in female CC026, CC027, and CC049 mice. The washout effect was significant in most strains in both sexes (Figure 4).

In genomic DNA isolated from lung samples, the significant differences among groups in EB-GII were observed in most male and female mice, except for CC027 males (Figure 5).

The amounts of *bis*-N7G-BD cross-links in genomic DNA are 10-fold lower as compared to EB-GII; these DNA lesions are more difficult to quantify, especially when using very small tissue amounts. Thus, we were only able to quantify *bis*-N7G-BD with confidence in the liver. Figure 6 shows liver *bis*-N7G-BD data across strains, sexes, and treatment conditions. We observed significant effects of BD exposure and washout in both male and female CC079 mice. Adduct levels increased in other strains and both sexes for all exposure groups, but due to large variability within each group, the effects were not statistically significant for the other four strains.

**Figure 7:**
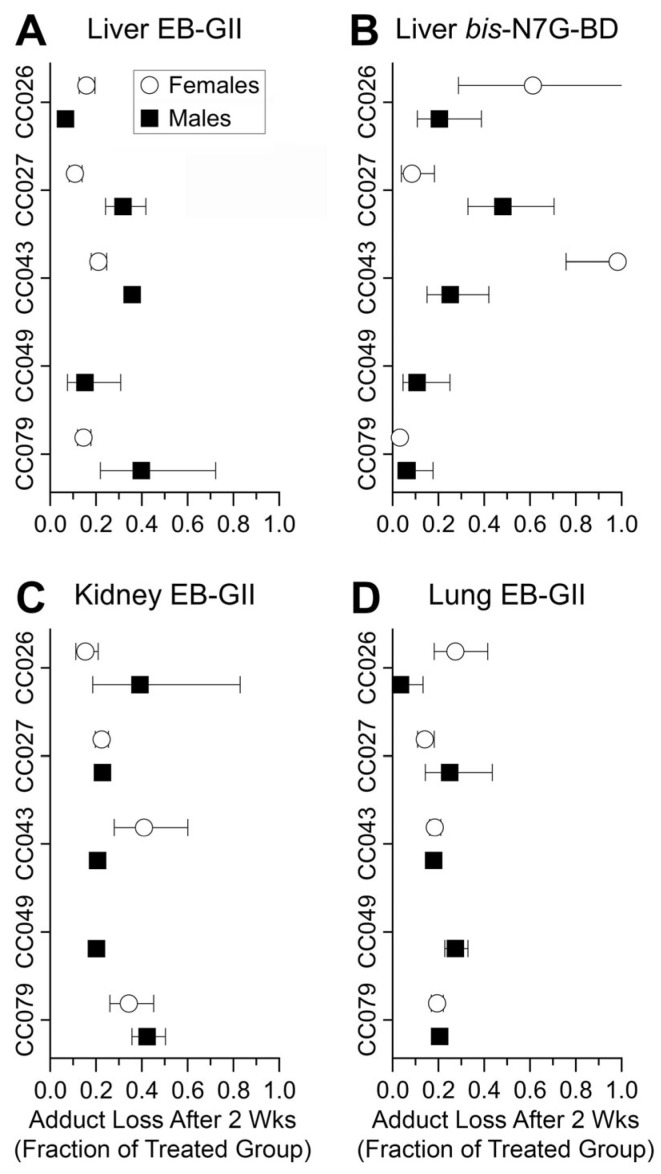
Fraction of BD adducts remaining in genomic DNA of mice subjected to BD treatment (600 ppm for 2 weeks) following a washout period (animals were left unexposed for 2 weeks after BD treatment) per tissue and strain/sex. Data for female mice are shown as open circles and data for male mice are shown as filled squares. Shown are group mean ± SD (*n* = 3–4/group). No samples were available for CC049 female mice.

To examine the rate of adduct loss from genomic DNA, we plotted the fraction of the adducts remaining in each tissue, strain, and sex 2 weeks following BD exposure (Figure 7). These data show that in nearly all strains and both sexes, the levels of EB-GII and *bis*-N7G-BD declined by 40 to 90% in 2 weeks after exposure to BD was discontinued. One exception was liver *bis*-N7G-BD in females from CC043—where minimal loss of this adduct was observed even after 2 weeks of recovery (Figure 7B).

**Figure 8:**
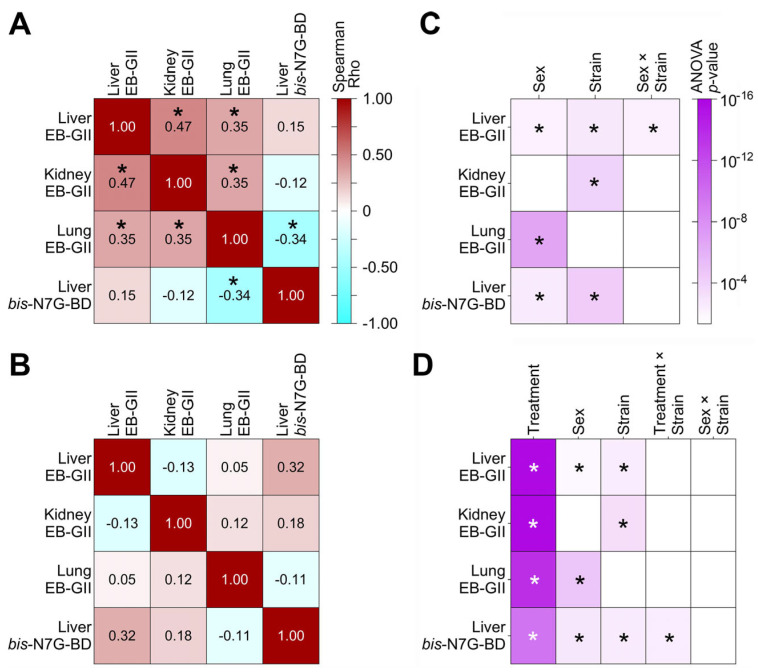
Correlations between adduct levels among tissues and sex and strain effects of BD exposure on DNA adduct levels. (**A**,**B**) Spearman correlations are shown as numbers and a heatmap (see inset in panel (**A**) for treated (1,3-butadiene, 600 ppm for 2 weeks), and washout (**B**) (left unexposed for 2 weeks after 1,3-butadiene treatment) animals). Asterisks (*) indicate significant (two-tailed, multiple-comparison-corrected *p* < 0.0332) correlations. (**C**,**D**) Significance levels for sex, strain, and interaction effects are shown as heatmaps from ANOVA analyses on the level/change in each adduct in liver, kidney, or lung (* highlights significance at the 0.05 level after multiple comparisons). Panel (**C**) depicts significance of effects for mice in the BD-exposed condition. Panel (**D**) depicts the significance of effects across the BD-exposed and washout conditions.

We next determined whether adduct levels in different tissues were correlated. Figure 8A,B show the results separately for the 2 week treatment (Figure 8A) and the washout groups (Figure 8B). In treated animals, a significant positive correlation was found for EB-GII adducts among all three tissues, although a significant negative correlation was found for lung EB-GII and liver *bis*-N7G-BD. In the washout groups, no significant correlations were observed, only marginal significance (*p* = 0.057) was found for liver EB-GII and *bis*-N7G-BD.

In addition, we tested for sex, strain, and interaction effects for each adduct and every tissue (Figure 8C,D). Panel C shows the significance of these effects in the BD exposed condition only. For the two BD adducts in the liver, adduct levels in males exceeded those in females, and for EB-GII in the lungs, levels were greater in females. Significant strain effects were observed in the liver and kidney for EB-GII, and in the liver for *bis*-N7G-BD. A significant sex × strain interaction was observed for EB-GII in the liver. Panel D shows the significance of these effects in the BD exposed and washout conditions, so that the effects of recovery across sexes and strains could be examined. All adducts/tissues showed a significant decline in adduct levels after a 2 week recovery period (Figure 7). The significance of main effects of sex and strain were consistent with those shown in Panel D. Finally, *bis*-N7G-BD levels in the liver showed a significant treatment × strain interaction, reflecting varying recoveries by strain, with strain CC079 showing the greatest recovery magnitude compared to other strains.

To translate our quantitative data on the population variability of BD-induced DNA adduct levels into information that is directly applicable to chemical risk assessment, each of the data sets was used to calculate chemical-specific uncertainty factors for human variability UF_H_ (Table 1). Interestingly, we found that the inter-strain variation in tissue levels of DNA adducts in CC mice was relatively modest after accounting for intra-strain variability. All the resulting UF_H_ values were less than the default value of 10-fold intended to address combined toxicokinetic and toxicodynamic variability, although in a few instances, the upper range of the confidence intervals exceeded 10. The largest UF_H_ value was for liver *bis*-N7G-BD at a protection level of 99% of the population, resulting in a factor of 5.85 [95% CI: 2.19–36.57]. By contrast, the values of UF_H_ derived from the EB-GII data were lower, all below 2.5.

## 4. Discussion

BD is a potent industrial and environmental carcinogen with widespread exposure to human populations. Due to potential inter-individual differences in risk following exposure to BD, studies of the dynamics of BD DNA adduct formation and persistence are needed [3,4,5,6,7,8]. Accordingly, this investigation focused on several aspects of remaining unknowns in BD DNA damage across tissues, sexes, and strains—(i) a comparative analysis of structurally distinct BD DNA adducts, (ii) determining the persistence of BD-induced DNA adducts in vivo, and (iii) quantifying inter-strain variability in BD DNA adduct formation and repair.

In this study, we focused on two BD adducts: N-7-(1-hydroxy-3-buten-2-yl) guanine (EB-GII) and 1,4-bis-(guan-7-yl)-2,3-butanediol (*bis*-N7G-BD). EB-GII is formed when BD metabolite 3,4-epoxy-1-butene (EB) reacts with the N7 position of guanine in DNA. This reaction is a major pathway for BD-induced DNA damage in vivo, leading to relatively high levels of EB-GII adducts in various tissues of exposed rats and mice. The observed EB-GII levels reported herein for mice exposed to comparably high amounts of BD are nearly identical to previous publications. By contrast, *bis*-N7G-BD adducts are DNA–DNA crosslinks that are formed by the diepoxide metabolite of BD (DEB) that can alkylate guanine bases in the opposite strands of DNA. DEB is a less abundant metabolite of BD compared to EB [26]. As a result, the number of *bis*-N7G-BD crosslinks in BD-treated animals is 10 times lower than that of EB-GII adducts. In our study, similar differences were observed between adducts, with values for *bis*-N7G-BD averaging 0.45 × 10^7^ nucleotides in the liver, and tissue-specific EB-GII values ranging from 0.97 to 2.16 × 10^6^ nucleotides (Table 1).

Our results revealed large inter-individual variability in genomic levels of BD-induced EB-GII and *bis*-N7G-BD adducts in tissues of BD-exposed animals, commensurate with previous observations for other BD adducts. EB-GII has previously been proposed as a specific biomarker of exposure to BD [27] because it is not formed endogenously. EB-GII adducts themselves are considered to have low intrinsic mutagenic potency because the N7 position of guanine does not participate in Watson–Crick–Franklin base pairing and thus these adducted guanines still retain their ability to pair with cytosine [27]. By contrast, N7-guanine crosslinks are much more mutagenic than EB-GII because of their cross-linked structure that fuses DNA strands and hinders replication [28]. As a result, DEB, the precursor to these crosslinks, is the most mutagenic BD metabolite, with mutagenic potency that is up to 200-fold higher than that for EB [13]. DNA–DNA crosslinks are considered critical lesions in BD-induced carcinogenesis because they both block DNA replication and induce error-prone repair, leading to point mutations, large deletions, and chromosomal aberrations [29,30]. Thus, it is noteworthy that while we observed that tissue levels of these adducts were largely concordant with previous reports, the present study provides important new information regarding BD DNA adduct levels in different tissues and quantifies the degree of inter-strain variability, adding important information to both mechanistic considerations and the quantitative risk assessment of BD.

We found that genomic levels of EB-GII in BD-exposed mice showed significant correlation among tissues. By contrast, liver levels of *bis*-N7G-BD were significantly negatively correlated with lung EB-GII and did not correlate with EB-GII in the liver or kidneys (Figure 8A). In addition, liver *bis*-N7G-BD levels showed the highest inter-strain variability. Interestingly, the strain effect was significant for both EB-GII and *bis*-N7G-BD apart from lung EB-GII (Figure 8D). Therefore, based on these data taken together, we reason that in future studies of BD effects, these two DNA adducts, EB-GII and *bis*-N7G-BD, may be the most sensible endpoints for evaluation based on their known mechanistic (i.e., effect) and biomarker (i.e., exposure) value and non-redundant nature. Still, because of the very low abundance of *bis*-N7G-BD, the analytical method sensitivity remains a challenge, especially in studies where low amounts of tissues/cells are available.

With respect to sex differences in BD DNA adducts, it is well established in animal models that females often show higher levels of DNA adducts, crosslinks, and mutagenicity, particularly in the liver [13]. These differences are influenced by metabolic, genetic, and epigenetic factors, and may underlie observed sex disparities in cancer susceptibility. Indeed, our data confirm that BD DNA adduct levels were generally higher in females. In addition, the sex effect was significant for both EB-GII and *bis*-N7G-BD, and the highest significance was observed for lung EB-GII levels. However, strain × sex effects were significant only for liver EB-GII.

Another noteworthy observation in the current study is the difference in persistence of BD DNA adducts after exposure was terminated. It was found that the half-lives of *bis*-N7G-BD in mouse liver, kidney, and lungs were between 2.5 and 5 days, respectively. In vitro, the half-life of *bis*-N7G-BD was ~4 days due to spontaneous depurination. In the present study, the fraction of liver *bis*-N7G-BD remaining 2 weeks post-exposure was 20%, which is consistent with the previously reported in vivo half-life.

Finally, our study addresses one of the most persistent information gaps regarding BD risk assessment—quantitative characterization of inter-individual variability. The original inhalation risk assessment of BD is decades old and the US EPA is currently finalizing updated reassessment [11]. Still, even though data on molecular biomarkers of exposure and effect are now available from both human studies of workers and experimental studies in rodents, few attempts have been made to quantitatively evaluate the variability in adverse effects of BD in either humans or animals. Available human data suggests that a default uncertainty factor of 10 for inter-individual variability (UF_H_) may be marginally protective because greater variability was reported for some biomarkers. For example, it was more than 10-fold for EB-GII [16], but for blood THB-Val, it was ~3-fold. Animal studies in population-based designs provide additional information in support of the choice for UF_H_. For example, the experimentally derived UF_H_ were generally no more than 2-fold for *N*-7-(2,3,4-trihydroxybut-1-yl)-guanine (THB-Gua) adducts in a study of a mouse CC population exposed to BD at a similar dose and duration [23]. Another mouse CC study examined BD hemoglobin adducts and showed the range from 2 to 7.51, depending on the dose and the adduct. In a previous report of urinary EB-GII adducts from the animals used in the current study, we found low variability with UF_H_ for the 99th percentile ranging from 2.0 in males to 3.1 in females [16]. In the current study, we performed similar analyses for EB-GII, and *bis*-N7G-BD adducts in genomic DNA across mouse tissues and the UF_H_ for the 99th percentile ranged from 1.4 to 2.2 for EB-GII. The greatest variability was observed for *bis*-N7G-BD with a UF_H_ of 5.9 (Table 1). Collectively, we found that studies in mice show that an UF_H_ of 10 may be scientifically justifiable based on the available body of evidence listed above. There are several limitations of our mouse study. One is that, for practical reasons, we employed high doses of BD where saturation of metabolic activation is a known phenomenon [13]. Another is that our study extended 2 weeks, which is far shorter than the time required for tumor formation [31]. Therefore, additional studies are needed to test effects of extended and more human-relevant BD exposures; however, even more sensitive analytical methods for DNA adducts would also be necessary to enable such investigations.

## 5. Conclusions

This study compared the concentrations of two major BD-induced DNA adducts—EB-GII and *bis*-N7G-BD—across tissues, sexes, and mouse strains, revealing that EB-GII was more abundant than *bis*-N7G-BD and that both adducts were significantly variable by sex and strain. Genomic EB-GII levels correlated across tissues and showed moderate inter-strain variation, whereas bis-N7G-BD in the liver showed high inter-strain variability and was negatively correlated with lung EB-GII. Females generally exhibited higher adduct levels, especially in the liver, and sex significantly influenced the levels of most adducts. Persistence studies showed that both adducts are relatively short-lived, but EB-GII’s persistence varied by sex and strain, suggesting additional removal mechanisms beyond spontaneous depurination. The study also highlighted that inter-individual variability in adduct levels is generally low, supporting the use of a 10-fold uncertainty factor in human risk assessment, though this is based on high-dose exposures in animals.

## Figures and Tables

**Table 1:** Quantitation of variability (within and across strains) in tissue levels of BD -DNA adducts following 1,3-Butadiene exposure in CC mice (corrected for sex effects). (CI, confidence interval; GM, geometric mean; GSD, geometric standard deviation; σ^2^ (total), variance of log-transformed adduct levels; σ^2^ (within), variance of log-transformed adduct levels within strain; σ^2^ (across), variance of log-transformed adduct levels across strains; UF_H_ (95%), human variability factor for the 95th percentile relative to the median; UF_H_ (99%), human toxicokinetic variability factor for the 99th percentile relative to the median).

		Exposure	Washout
Liver EB-GII (per 10^6^ nucleotides)	GM uncorrected (95% CI)	2.0 (0.46–8.77)	0.35 (0.14–0.86)
	GSD uncorrected (95% CI)	2.08 (1.83–2.52)	1.56 (1.44–1.78)
	σ^2^ (total)	0.54	0.2
	σ^2^ (within)	0.42	0.19
	σ^2^ (across)	0.12	0.01
	UF_H_ (95% CI)	1.77 (1.09–3.47)	1.18 (1–1.7)
	UF_H_ (99% CI)	2.24 (1.13–5.8)	1.27 (1–2.12)
Liver *bis*-N7G-BD (per 10^7^ nucleotides)	GM uncorrected (95% CI)	0.45 (0.04–4.54)	0.09 (0.01–1.13)
	GSD uncorrected (95% CI)	3.14 (2.58–4.25)	3.54 (2.81–5.1)
	σ^2^ (total)	1.31	1.6
	σ^2^ (within)	0.74	1.46
	σ^2^ (across)	0.58	0.14
	UF_H_ (95% CI)	3.49 (1.74–12.74)	1.86 (1–5.4)
	UF_H_ (99% CI)	5.85 (2.19–36.57)	2.4 (1–10.86)
Lung EB-GII (per 10^6^ nucleotides)	GM uncorrected (95% CI)	2.16 (0.72–6.46)	0.35 (0.03–4.46)
	GSD uncorrected (95% CI)	1.72 (1.57–1.99)	3.54 (2.81–5.1)
	σ^2^ (total)	0.3	1.6
	σ^2^ (within)	0.27	1.6
	σ^2^ (across)	0.02	0
	UF_H_ (95% CI)	1.27 (1–1.81)	1.0 (1.0–2.34)
	UF_H_ (99% CI)	1.41 (1–2.31)	1.0 (1.0–3.33)
Kidney EB-GII (per 10^6^ nucleotides)	GM uncorrected (95% CI)	0.97 (0.35–2.68)	0.27 (0.06–1.11)
	GSD uncorrected (95% CI)	1.65 (1.52–1.89)	2.02 (1.78–2.48)
	σ^2^ (total)	0.25	0.5
	σ^2^ (within)	0.14	0.46
	σ^2^ (across)	0.11	0.04
	UF_H_ (95% CI)	1.74 (1.27–3.11)	1.37 (1–2.45)
	UF_H_ (99% CI)	2.19 (1.4–4.98)	1.56 (1–3.55)

## Data Availability

Adduct data are included as Table S1.

## Supplementary Materials

The following supporting information can be downloaded at https://www.mdpi.com/article/10.3390/toxics13100844/s1. Table S1: Adduct levels in liver, kidney and lung of CC mice exposed to BD. Figure S1: Validation curves of EB-GII/bis-N7G-BD. Standard was spiked into salmon sperm DNA and subjected to normal sample preparation including neutral thermal hydrolysis, HPLC offline purification, and nano-LC/MS analysis. LOD/LOQ determined by (slope error * X)/slope where X = 3.3 (LOD) or 10 (LOQ). Figure S2: Example offline HPLC chromatogram for EBGII/bis-N7G-BD in mouse kidney. Highlighted regions from 15.5–18.0 min were collected and concentrated for nano-LC/MS analysis.

## Abbreviations

The following abbreviations are used in this manuscript:

BD	1,3-butadiene
EB	3,4-epoxy-1-butene
DEB	1,2,3,4
EB-GII	N7-(1-hydroxyl-3-buten-1-yl)guanine
*bis-*N7G-BD	1,4-bis-(guan-7-yl)-2,3-butanediol
CC	Collaborative Cross
HPLC-ESI-MS/MS	High-Performance Liquid chromatography-electrospray ionization-mass spectrometry/mass spectrometry
dG	deoxyguanosine
FAPy	formamidopyrimidine
