# Supplementary material for: Strain and Sex Variability in Liver, Kidney and Lung Levels of DNA Adducts EB-GII and bis-N7G-BD Following Inhalation Exposure to 1,3-Butadiene in Collaborative Cross Mice
Source: Toxics. 2025 Oct 3;13(10):844. doi: 10.3390/toxics13100844 (PMC12568132; doi:10.3390/toxics13100844)
Supplement: Supplementary file 1 [file toxics-13-00844-s001.zip › toxics-3818555-supplementary.pdf]

toxics

MDPI

Supplementary Materials: Strain and Sex Variability in Liver, Kidney and Lung Levels of DNA Adducts EB-GII and *bis*-N7G-BD Following Inhalation Exposure to 1,3-Butadiene in Collaborative Cross Mice

Erik Moran, Samantha Goodman, Fred A. Wright, Richard Evans, Natalia Y. Tretyakova, and Ivan Rusyn

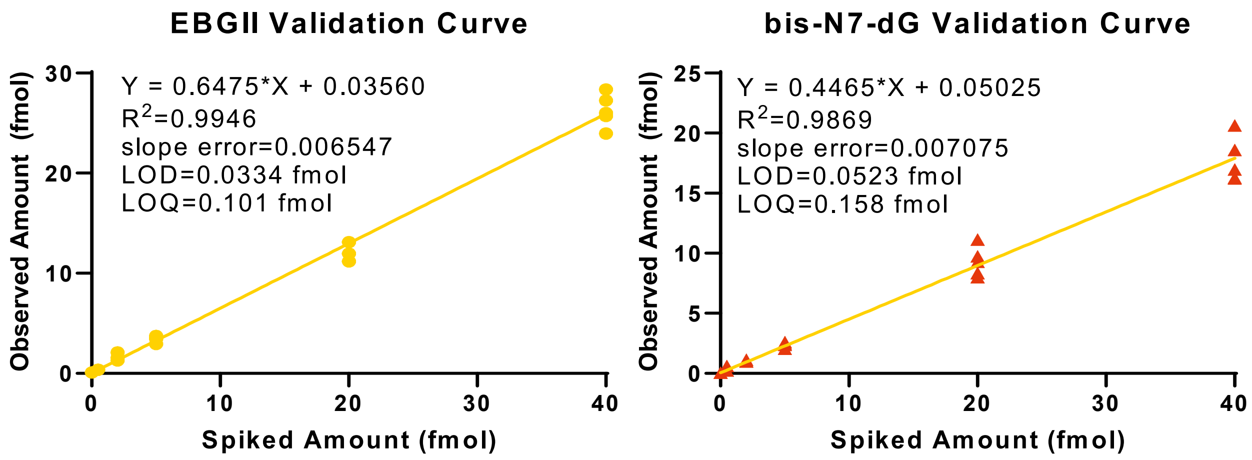

Figure S1. Validation curves of EB-GII/*bis*-N7G-BD. Standard was spiked into salmon sperm DNA and subjected to normal sample preparation including neutral thermal hydrolysis, HPLC offline purification, and nano-LC/MS analysis. LOD/LOQ determined by (slope error*X)/slope where X=3.3 (LOD) or 10 (LOQ).

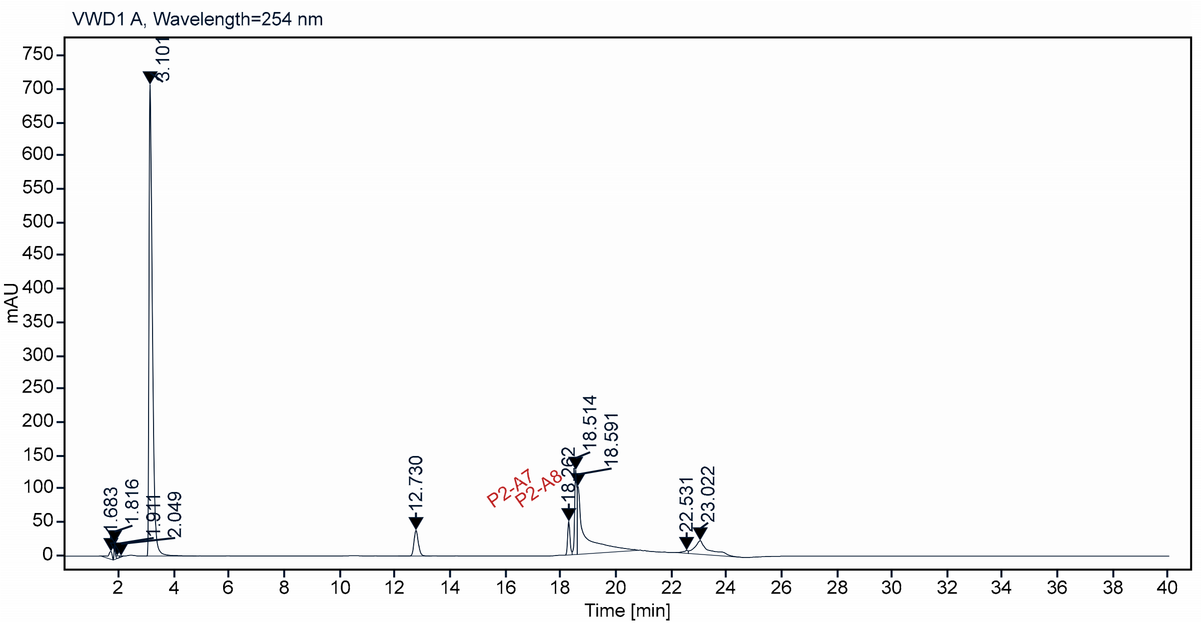

Figure S2. Example offline HPLC chromatogram for EBGII/*bis*-N7G-BD in mouse kidney. Highlighted regions from 15.5-18.0 min were collected and concentrated for nano-LC/MS analysis.
